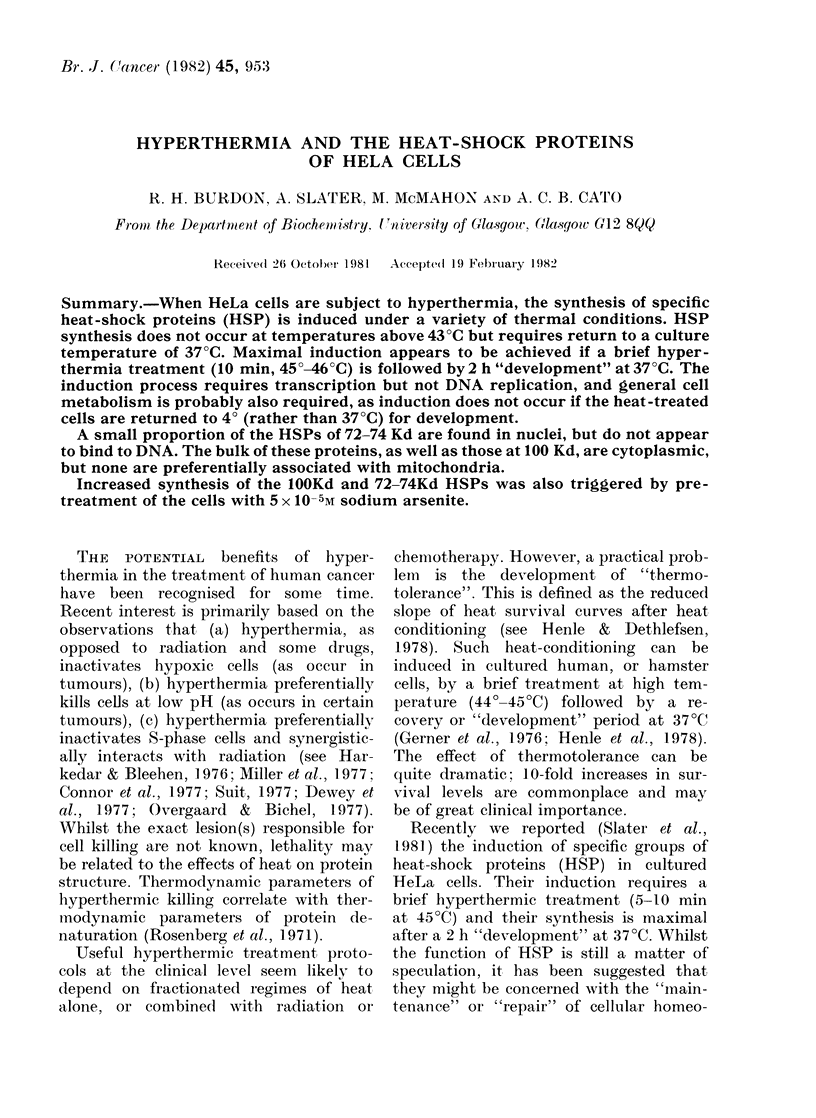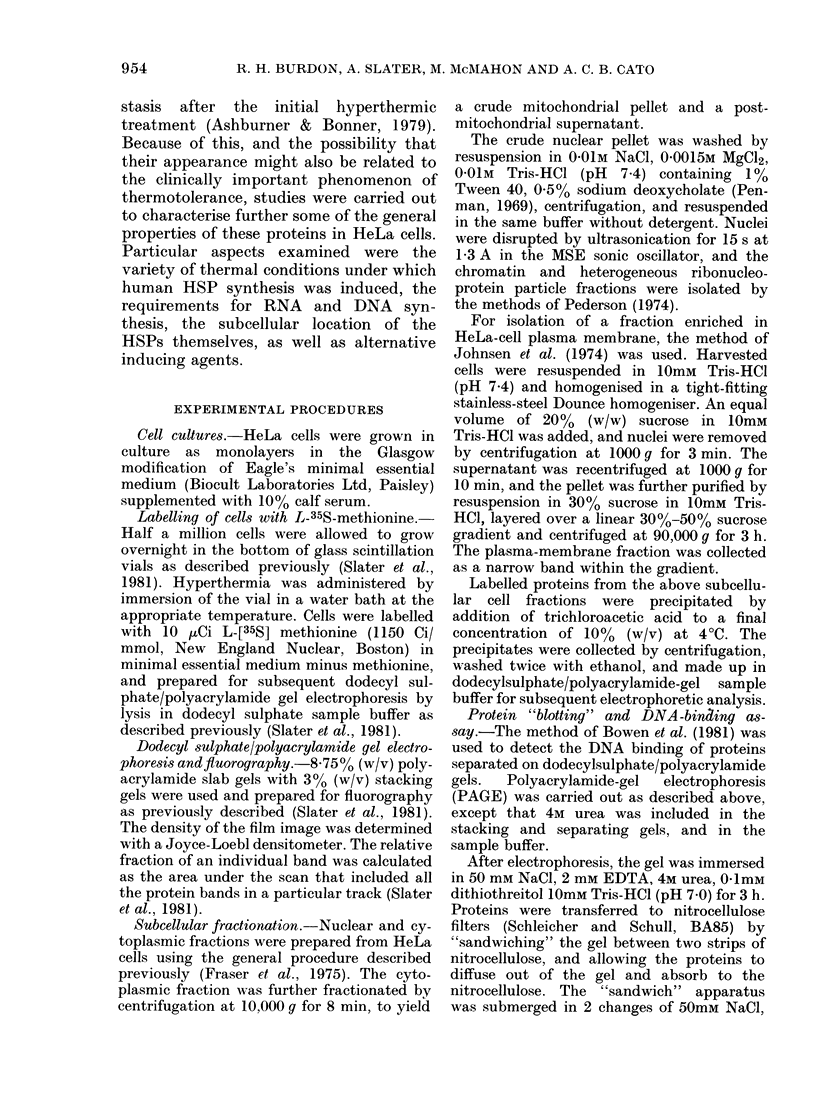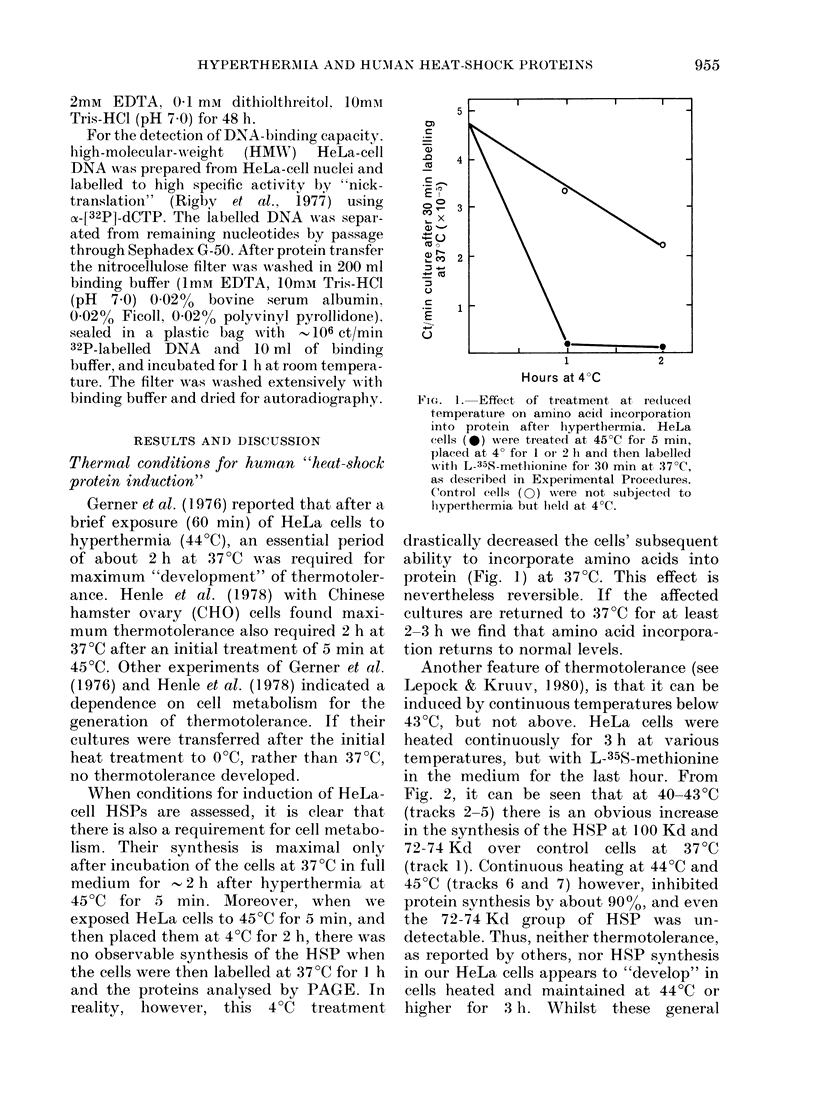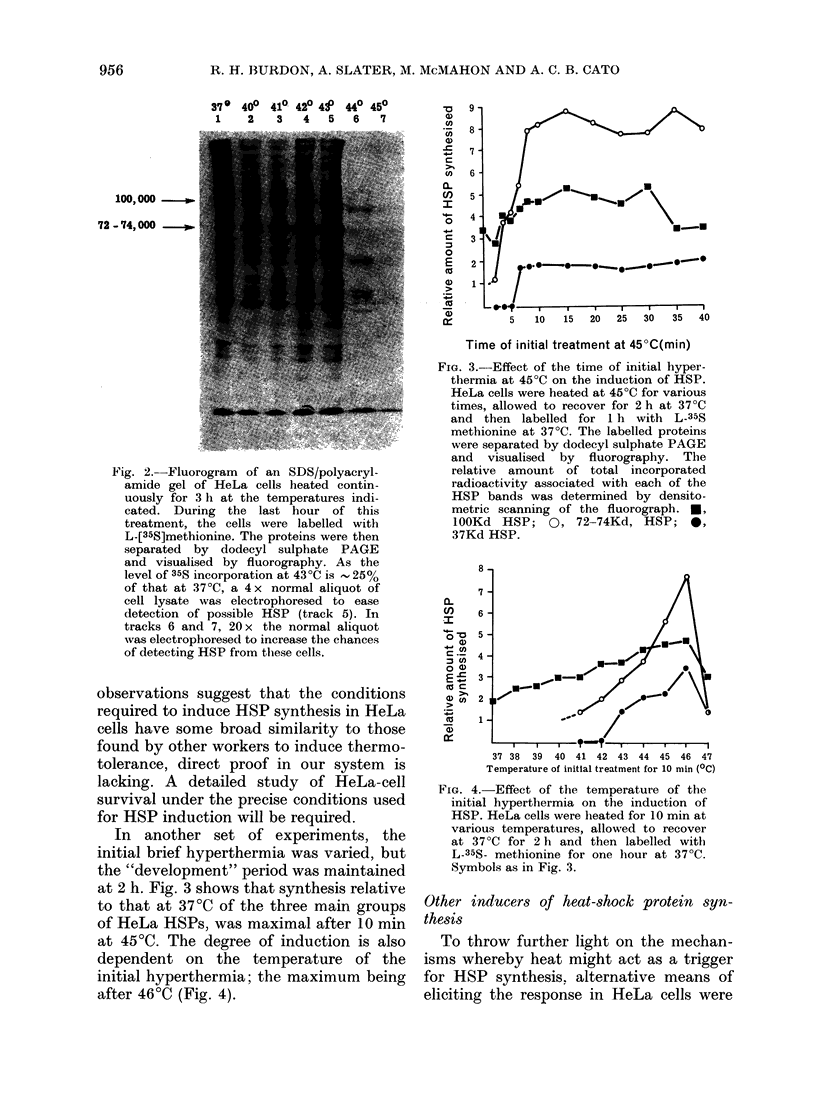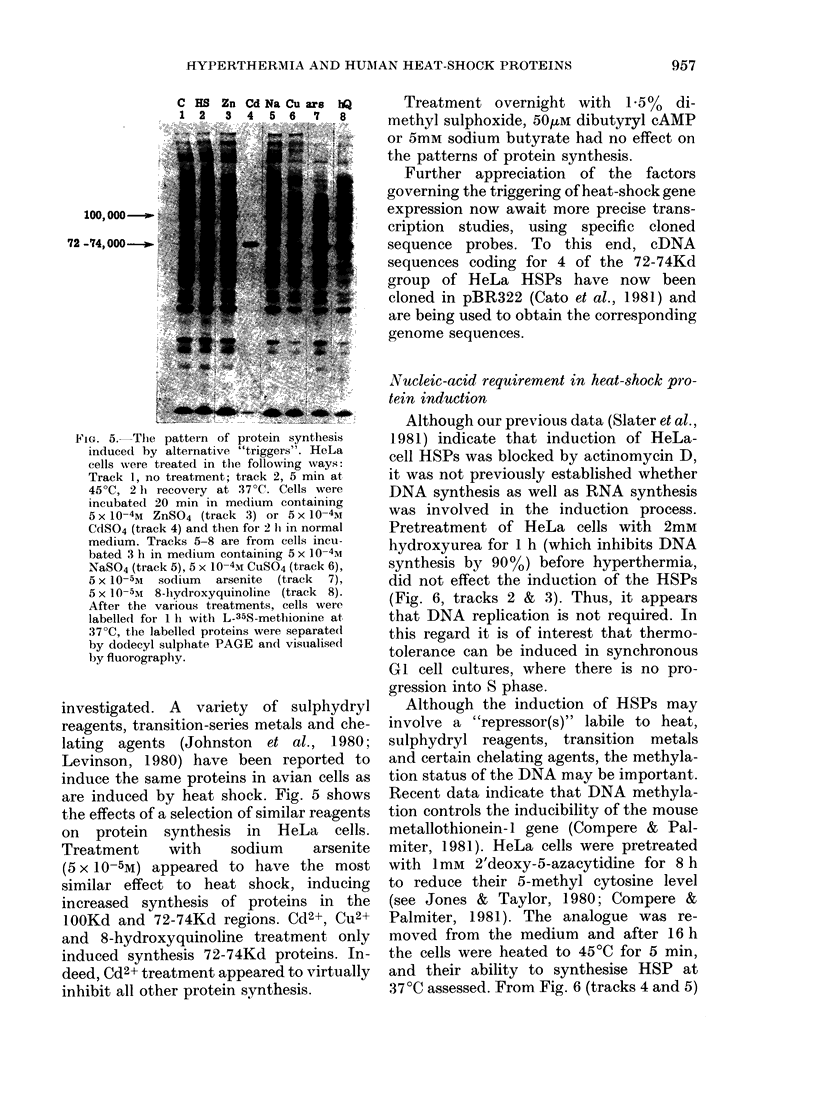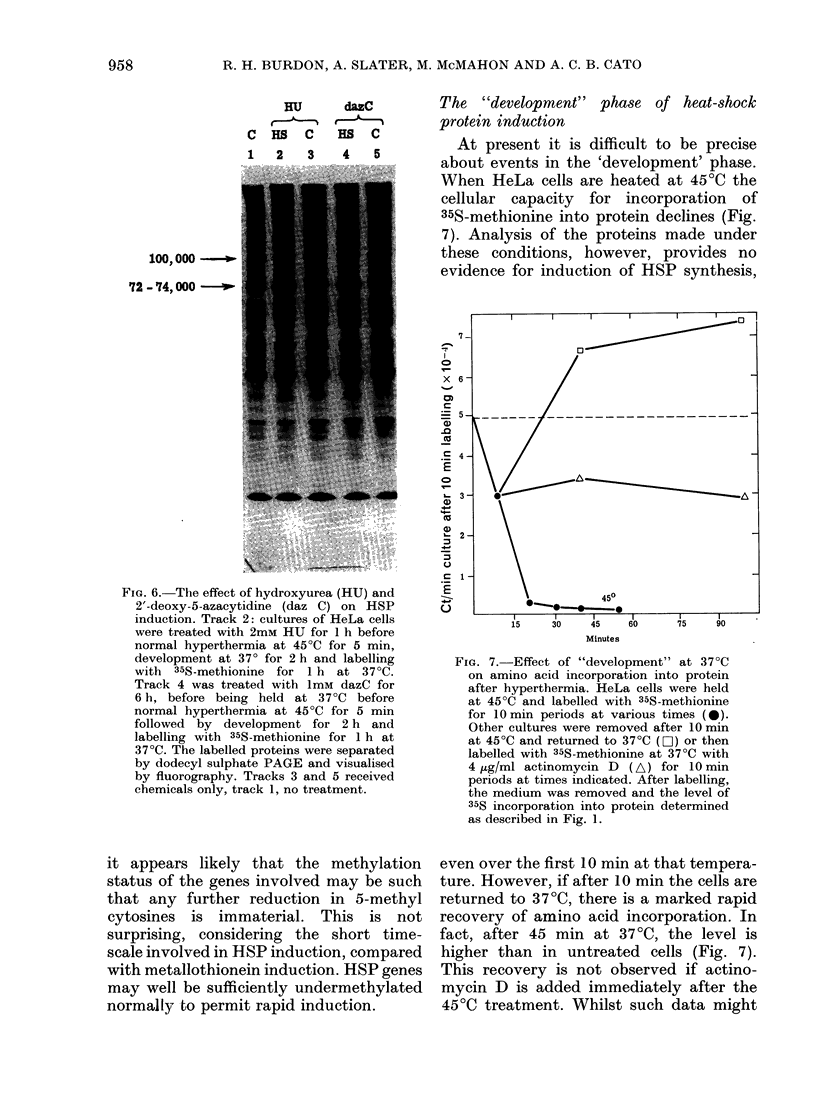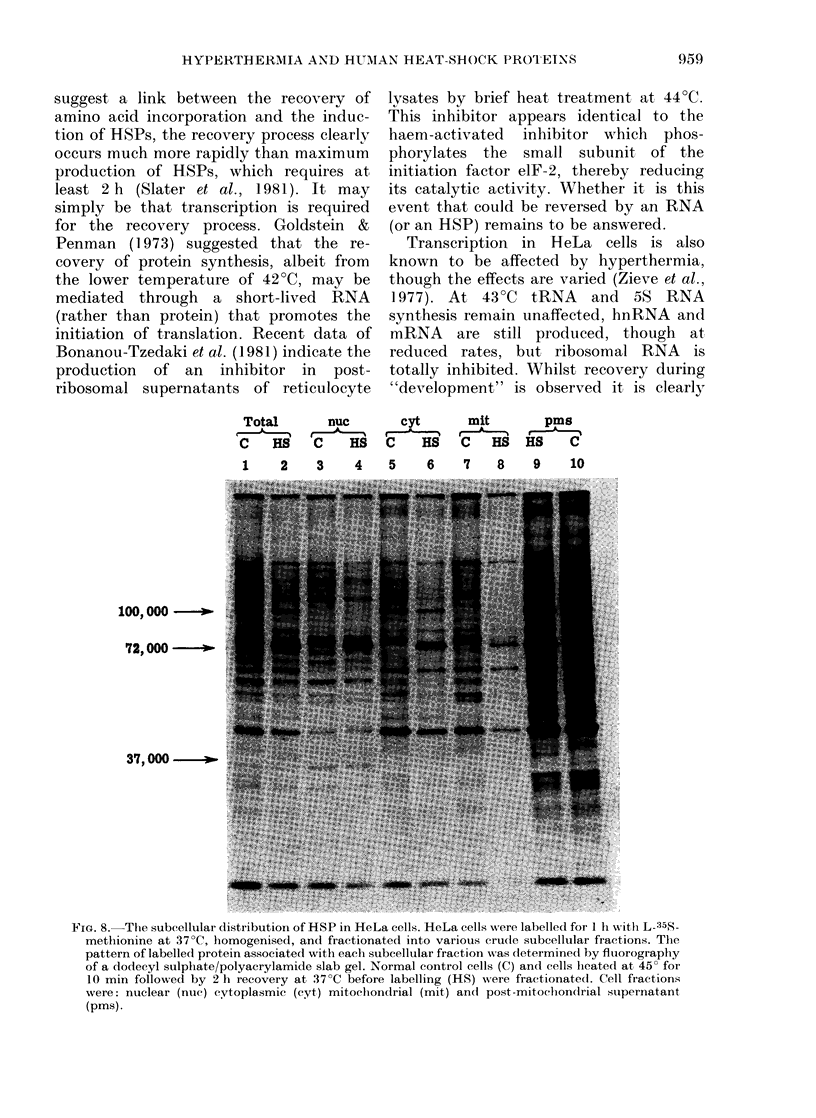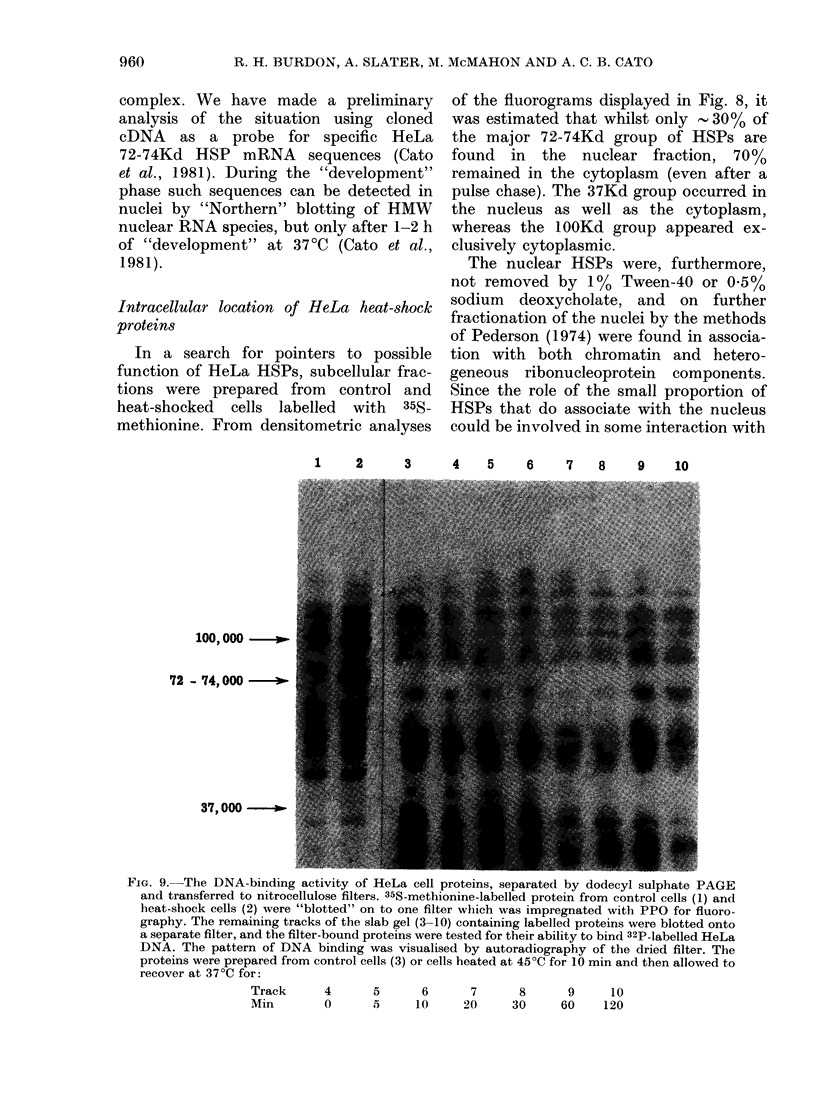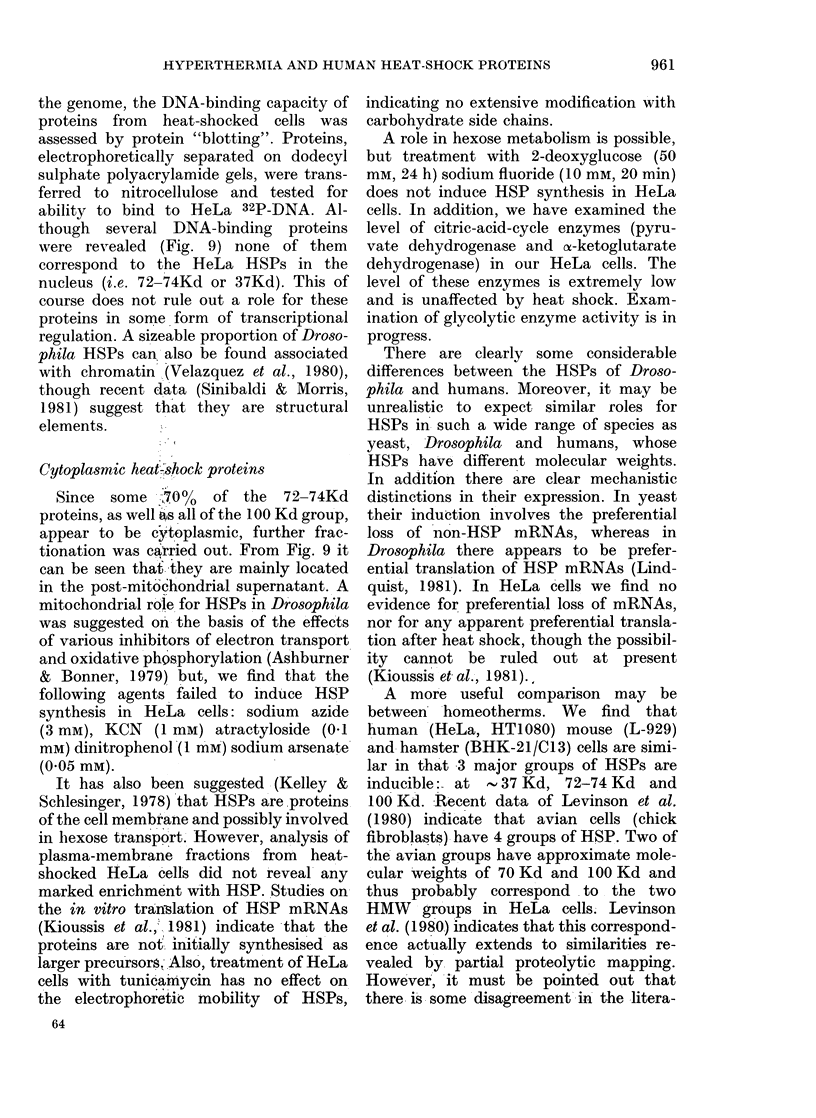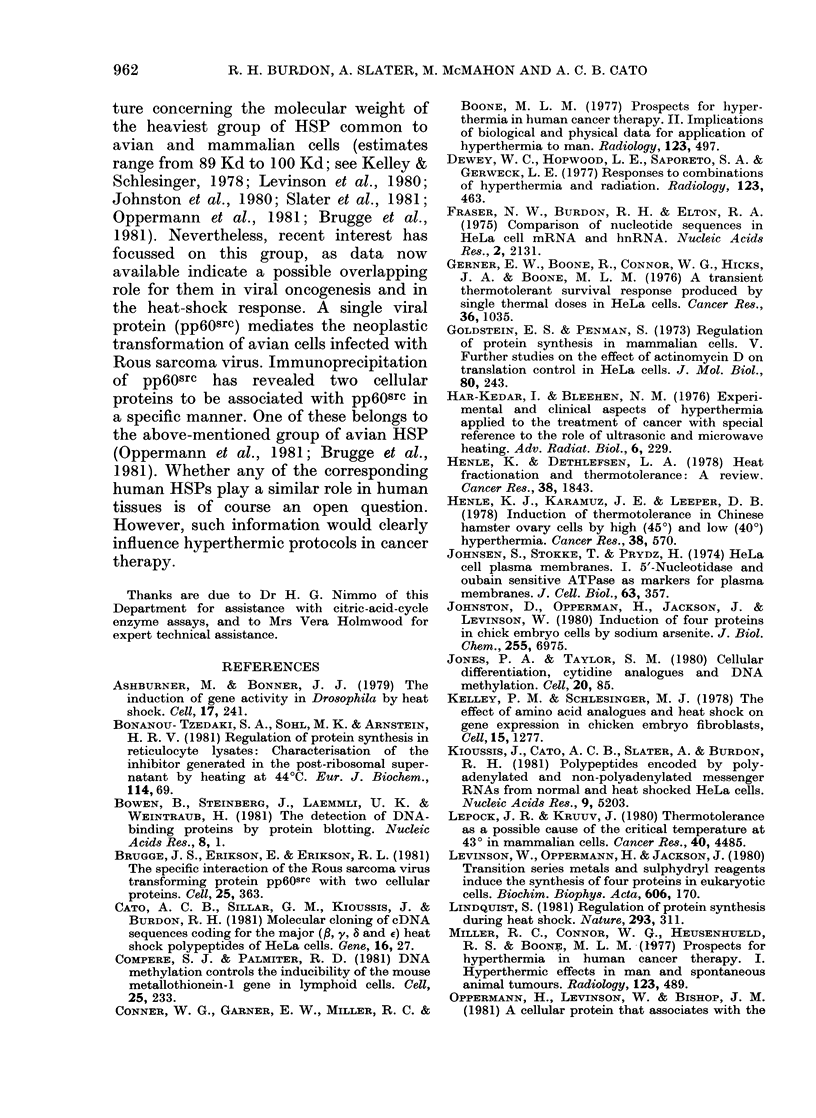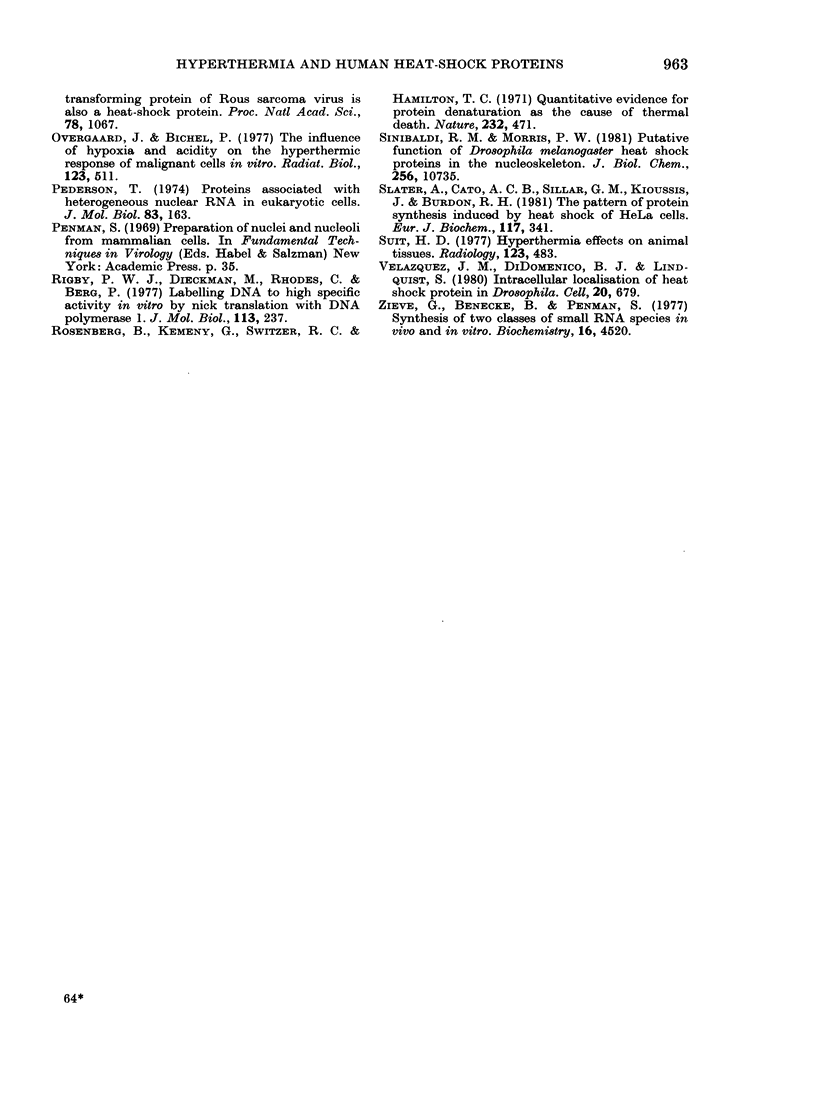# Hyperthermia and the heat-shock proteins of HeLa cells.

**DOI:** 10.1038/bjc.1982.148

**Published:** 1982-06

**Authors:** R. H. Burdon, A. Slater, M. McMahon, A. C. Cato

## Abstract

**Images:**


*Br. J. Cancer* (1982) **45**, 953

### HYPERTHERMIA AND THE HEAT-SHOCK PROTEINS OF HELA CELLS

R. H. BURDON, A. SLATER, M. McMAHON AND A. C. B. CATO

*From the Department of Biochemistry, University of Glasgow, Glasgow G12 8QQ*

Received 26 October 1981 Accepted 19 February 1982

**Summary.—When HeLa cells are subject to hyperthermia, the synthesis of specific heat-shock proteins (HSP) is induced under a variety of thermal conditions. HSP synthesis does not occur at temperatures above 43°C but requires return to a culture temperature of 37°C. Maximal induction appears to be achieved if a brief hyperthermia treatment (10 min, 45°–46°C) is followed by 2 h "development" at 37°C. The induction process requires transcription but not DNA replication, and general cell metabolism is probably also required, as induction does not occur if the heat-treated cells are returned to 4° (rather than 37°C) for development.**

**A small proportion of the HSPs of 72–74 Kd are found in nuclei, but do not appear to bind to DNA. The bulk of these proteins, as well as those at 100 Kd, are cytoplasmic, but none are preferentially associated with mitochondria.**

**Increased synthesis of the 100Kd and 72–74Kd HSPs was also triggered by pretreatment of the cells with $5 \times 10^{-5}$M sodium arsenite.**

THE POTENTIAL benefits of hyperthermia in the treatment of human cancer have been recognised for some time. Recent interest is primarily based on the observations that (a) hyperthermia, as opposed to radiation and some drugs, inactivates hypoxic cells (as occur in tumours), (b) hyperthermia preferentially kills cells at low pH (as occurs in certain tumours), (c) hyperthermia preferentially inactivates S-phase cells and synergistically interacts with radiation (see Harkedar & Bleehen, 1976; Miller *et al.*, 1977; Connor *et al.*, 1977; Suit, 1977; Dewey *et al.*, 1977; Overgaard & Bichel, 1977). Whilst the exact lesion(s) responsible for cell killing are not known, lethality may be related to the effects of heat on protein structure. Thermodynamic parameters of hyperthermic killing correlate with thermodynamic parameters of protein denaturation (Rosenberg *et al.*, 1971).

Useful hyperthermic treatment protocols at the clinical level seem likely to depend on fractionated regimes of heat alone, or combined with radiation or chemotherapy. However, a practical problem is the development of "thermotolerance". This is defined as the reduced slope of heat survival curves after heat conditioning (see Henle & Dethlefsen, 1978). Such heat-conditioning can be induced in cultured human, or hamster cells, by a brief treatment at high temperature (44°–45°C) followed by a recovery or "development" period at 37°C (Gerner *et al.*, 1976; Henle *et al.*, 1978). The effect of thermotolerance can be quite dramatic; 10-fold increases in survival levels are commonplace and may be of great clinical importance.

Recently we reported (Slater *et al.*, 1981) the induction of specific groups of heat-shock proteins (HSP) in cultured HeLa cells. Their induction requires a brief hyperthermic treatment (5–10 min at 45°C) and their synthesis is maximal after a 2 h "development" at 37°C. Whilst the function of HSP is still a matter of speculation, it has been suggested that they might be concerned with the "maintenance" or "repair" of cellular homeostasis after the initial hyperthermic treatment (Ashburner & Bonner, 1979). Because of this, and the possibility that their appearance might also be related to the clinically important phenomenon of thermotolerance, studies were carried out to characterise further some of the general properties of these proteins in HeLa cells. Particular aspects examined were the variety of thermal conditions under which human HSP synthesis was induced, the requirements for RNA and DNA synthesis, the subcellular location of the HSPs themselves, as well as alternative inducing agents.

### EXPERIMENTAL PROCEDURES

*Cell cultures.*—HeLa cells were grown in culture as monolayers in the Glasgow modification of Eagle's minimal essential medium (Biocult Laboratories Ltd, Paisley) supplemented with 10% calf serum.

*Labelling of cells with L-$^{35}$S-methionine.*—Half a million cells were allowed to grow overnight in the bottom of glass scintillation vials as described previously (Slater *et al.*, 1981). Hyperthermia was administered by immersion of the vial in a water bath at the appropriate temperature. Cells were labelled with 10 $\mu$Ci L-[$^{35}$S] methionine (1150 Ci/mmol, New England Nuclear, Boston) in minimal essential medium minus methionine, and prepared for subsequent dodecyl sulphate/polyacrylamide gel electrophoresis by lysis in dodecyl sulphate sample buffer as described previously (Slater *et al.*, 1981).

*Dodecyl sulphate/polyacrylamide gel electrophoresis and fluorography.*—8·75% (w/v) polyacrylamide slab gels with 3% (w/v) stacking gels were used and prepared for fluorography as previously described (Slater *et al.*, 1981). The density of the film image was determined with a Joyce-Loebl densitometer. The relative fraction of an individual band was calculated as the area under the scan that included all the protein bands in a particular track (Slater *et al.*, 1981).

*Subcellular fractionation.*—Nuclear and cytoplasmic fractions were prepared from HeLa cells using the general procedure described previously (Fraser *et al.*, 1975). The cytoplasmic fraction was further fractionated by centrifugation at 10,000 *g* for 8 min, to yield a crude mitochondrial pellet and a post-mitochondrial supernatant.

The crude nuclear pellet was washed by resuspension in 0·01M NaCl, 0·0015M $MgCl_2$, 0·01M Tris-HCl (pH 7·4) containing 1% Tween 40, 0·5% sodium deoxycholate (Penman, 1969), centrifugation, and resuspended in the same buffer without detergent. Nuclei were disrupted by ultrasonication for 15 s at 1·3 A in the MSE sonic oscillator, and the chromatin and heterogeneous ribonucleoprotein particle fractions were isolated by the methods of Pederson (1974).

For isolation of a fraction enriched in HeLa-cell plasma membrane, the method of Johnsen *et al.* (1974) was used. Harvested cells were resuspended in 10mM Tris-HCl (pH 7·4) and homogenised in a tight-fitting stainless-steel Dounce homogeniser. An equal volume of 20% (w/w) sucrose in 10mM Tris-HCl was added, and nuclei were removed by centrifugation at 1000 *g* for 3 min. The supernatant was recentrifuged at 1000 *g* for 10 min, and the pellet was further purified by resuspension in 30% sucrose in 10mM Tris-HCl, layered over a linear 30%–50% sucrose gradient and centrifuged at 90,000 *g* for 3 h. The plasma-membrane fraction was collected as a narrow band within the gradient.

Labelled proteins from the above subcellular cell fractions were precipitated by addition of trichloroacetic acid to a final concentration of 10% (w/v) at 4°C. The precipitates were collected by centrifugation, washed twice with ethanol, and made up in dodecylsulphate/polyacrylamide-gel sample buffer for subsequent electrophoretic analysis.

*Protein "blotting" and DNA-binding assay.*—The method of Bowen *et al.* (1981) was used to detect the DNA binding of proteins separated on dodecylsulphate/polyacrylamide gels. Polyacrylamide-gel electrophoresis (PAGE) was carried out as described above, except that 4M urea was included in the stacking and separating gels, and in the sample buffer.

After electrophoresis, the gel was immersed in 50 mM NaCl, 2 mM EDTA, 4M urea, 0·1mM dithiothreitol 10mM Tris-HCl (pH 7·0) for 3 h. Proteins were transferred to nitrocellulose filters (Schleicher and Schull, BA85) by "sandwiching" the gel between two strips of nitrocellulose, and allowing the proteins to diffuse out of the gel and absorb to the nitrocellulose. The "sandwich" apparatus was submerged in 2 changes of 50mM NaCl,

2mM EDTA, 0·1 mM dithiolthreitol, 10mM Tris-HCl (pH 7·0) for 48 h.

For the detection of DNA-binding capacity, high-molecular-weight (HMW) HeLa-cell DNA was prepared from HeLa-cell nuclei and labelled to high specific activity by "nick-translation" (Rigby *et al.*, 1977) using α-[$^{32}$P]-dCTP. The labelled DNA was separated from remaining nucleotides by passage through Sephadex G-50. After protein transfer the nitrocellulose filter was washed in 200 ml binding buffer (1mM EDTA, 10mM Tris-HCl (pH 7·0) 0·02% bovine serum albumin, 0·02% Ficoll, 0·02% polyvinyl pyrollidone), sealed in a plastic bag with ~$10^6$ ct/min $^{32}$P-labelled DNA and 10 ml of binding buffer, and incubated for 1 h at room temperature. The filter was washed extensively with binding buffer and dried for autoradiography.

FIG. 1.—Effect of treatment at reduced temperature on amino acid incorporation into protein after hyperthermia. HeLa cells (●) were treated at 45°C for 5 min, placed at 4° for 1 or 2 h and then labelled with L-$^{35}$S-methionine for 30 min at 37°C, as described in Experimental Procedures. Control cells (○) were not subjected to hyperthermia but held at 4°C.

### RESULTS AND DISCUSSION

### *Thermal conditions for human "heat-shock protein induction"*

Gerner *et al.* (1976) reported that after a brief exposure (60 min) of HeLa cells to hyperthermia (44°C), an essential period of about 2 h at 37°C was required for maximum "development" of thermotolerance. Henle *et al.* (1978) with Chinese hamster ovary (CHO) cells found maximum thermotolerance also required 2 h at 37°C after an initial treatment of 5 min at 45°C. Other experiments of Gerner *et al.* (1976) and Henle *et al.* (1978) indicated a dependence on cell metabolism for the generation of thermotolerance. If their cultures were transferred after the initial heat treatment to 0°C, rather than 37°C, no thermotolerance developed.

When conditions for induction of HeLa-cell HSPs are assessed, it is clear that there is also a requirement for cell metabolism. Their synthesis is maximal only after incubation of the cells at 37°C in full medium for ~2 h after hyperthermia at 45°C for 5 min. Moreover, when we exposed HeLa cells to 45°C for 5 min, and then placed them at 4°C for 2 h, there was no observable synthesis of the HSP when the cells were then labelled at 37°C for 1 h and the proteins analysed by PAGE. In reality, however, this 4°C treatment drastically decreased the cells' subsequent ability to incorporate amino acids into protein (Fig. 1) at 37°C. This effect is nevertheless reversible. If the affected cultures are returned to 37°C for at least 2–3 h we find that amino acid incorporation returns to normal levels.

Another feature of thermotolerance (see Lepock & Kruuv, 1980), is that it can be induced by continuous temperatures below 43°C, but not above. HeLa cells were heated continuously for 3 h at various temperatures, but with L-$^{35}$S-methionine in the medium for the last hour. From Fig. 2, it can be seen that at 40–43°C (tracks 2–5) there is an obvious increase in the synthesis of the HSP at 100 Kd and 72-74 Kd over control cells at 37°C (track 1). Continuous heating at 44°C and 45°C (tracks 6 and 7) however, inhibited protein synthesis by about 90%, and even the 72-74 Kd group of HSP was undetectable. Thus, neither thermotolerance, as reported by others, nor HSP synthesis in our HeLa cells appears to "develop" in cells heated and maintained at 44°C or higher for 3 h. Whilst these general

Fig. 2.—Fluorogram of an SDS/polyacrylamide gel of HeLa cells heated continuously for 3 h at the temperatures indicated. During the last hour of this treatment, the cells were labelled with L-[$^{35}$S]methionine. The proteins were then separated by dodecyl sulphate PAGE and visualised by fluorography. As the level of $^{35}$S incorporation at 43°C is $\sim$25% of that at 37°C, a 4× normal aliquot of cell lysate was electrophoresed to ease detection of possible HSP (track 5). In tracks 6 and 7, 20× the normal aliquot was electrophoresed to increase the chances of detecting HSP from these cells.

Fig. 3.—Effect of the time of initial hyperthermia at 45°C on the induction of HSP. HeLa cells were heated at 45°C for various times, allowed to recover for 2 h at 37°C and then labelled for 1 h with L-$^{35}$S methionine at 37°C. The labelled proteins were separated by dodecyl sulphate PAGE and visualised by fluorography. The relative amount of total incorporated radioactivity associated with each of the HSP bands was determined by densitometric scanning of the fluorograph. ■, 100Kd HSP; ○, 72–74Kd, HSP; ●, 37Kd HSP.

Fig. 4.—Effect of the temperature of the initial hyperthermia on the induction of HSP. HeLa cells were heated for 10 min at various temperatures, allowed to recover at 37°C for 2 h and then labelled with L-$^{35}$S- methionine for one hour at 37°C. Symbols as in Fig. 3.

observations suggest that the conditions required to induce HSP synthesis in HeLa cells have some broad similarity to those found by other workers to induce thermotolerance, direct proof in our system is lacking. A detailed study of HeLa-cell survival under the precise conditions used for HSP induction will be required.

In another set of experiments, the initial brief hyperthermia was varied, but the "development" period was maintained at 2 h. Fig. 3 shows that synthesis relative to that at 37°C of the three main groups of HeLa HSPs, was maximal after 10 min at 45°C. The degree of induction is also dependent on the temperature of the initial hyperthermia; the maximum being after 46°C (Fig. 4).

*Other inducers of heat-shock protein synthesis*

To throw further light on the mechanisms whereby heat might act as a trigger for HSP synthesis, alternative means of eliciting the response in HeLa cells were

FIG. 5.—The pattern of protein synthesis induced by alternative "triggers". HeLa cells were treated in the following ways: Track 1, no treatment; track 2, 5 min at 45°C, 2 h recovery at 37°C. Cells were incubated 20 min in medium containing $5 \times 10^{-4}$M $ZnSO_4$ (track 3) or $5 \times 10^{-4}$M $CdSO_4$ (track 4) and then for 2 h in normal medium. Tracks 5–8 are from cells incubated 3 h in medium containing $5 \times 10^{-4}$M $NaSO_4$ (track 5), $5 \times 10^{-4}$M $CuSO_4$ (track 6), $5 \times 10^{-5}$M sodium arsenite (track 7), $5 \times 10^{-5}$M 8-hydroxyquinoline (track 8). After the various treatments, cells were labelled for 1 h with L-$^{35}$S-methionine at 37°C, the labelled proteins were separated by dodecyl sulphate PAGE and visualised by fluorography.

investigated. A variety of sulphydryl reagents, transition-series metals and chelating agents (Johnston *et al.*, 1980; Levinson, 1980) have been reported to induce the same proteins in avian cells as are induced by heat shock. Fig. 5 shows the effects of a selection of similar reagents on protein synthesis in HeLa cells. Treatment with sodium arsenite ($5 \times 10^{-5}$M) appeared to have the most similar effect to heat shock, inducing increased synthesis of proteins in the 100Kd and 72-74Kd regions. $Cd^{2+}$, $Cu^{2+}$ and 8-hydroxyquinoline treatment only induced synthesis 72-74Kd proteins. Indeed, $Cd^{2+}$ treatment appeared to virtually inhibit all other protein synthesis.

Treatment overnight with 1·5% dimethyl sulphoxide, 50μM dibutyryl cAMP or 5mM sodium butyrate had no effect on the patterns of protein synthesis.

Further appreciation of the factors governing the triggering of heat-shock gene expression now await more precise transcription studies, using specific cloned sequence probes. To this end, cDNA sequences coding for 4 of the 72-74Kd group of HeLa HSPs have now been cloned in pBR322 (Cato *et al.*, 1981) and are being used to obtain the corresponding genome sequences.

*Nucleic-acid requirement in heat-shock protein induction*

Although our previous data (Slater *et al.*, 1981) indicate that induction of HeLa-cell HSPs was blocked by actinomycin D, it was not previously established whether DNA synthesis as well as RNA synthesis was involved in the induction process. Pretreatment of HeLa cells with 2mM hydroxyurea for 1 h (which inhibits DNA synthesis by 90%) before hyperthermia, did not effect the induction of the HSPs (Fig. 6, tracks 2 & 3). Thus, it appears that DNA replication is not required. In this regard it is of interest that thermotolerance can be induced in synchronous G1 cell cultures, where there is no progression into S phase.

Although the induction of HSPs may involve a "repressor(s)" labile to heat, sulphydryl reagents, transition metals and certain chelating agents, the methylation status of the DNA may be important. Recent data indicate that DNA methylation controls the inducibility of the mouse metallothionein-1 gene (Compere & Palmiter, 1981). HeLa cells were pretreated with 1mM 2′deoxy-5-azacytidine for 8 h to reduce their 5-methyl cytosine level (see Jones & Taylor, 1980; Compere & Palmiter, 1981). The analogue was removed from the medium and after 16 h the cells were heated to 45°C for 5 min, and their ability to synthesise HSP at 37°C assessed. From Fig. 6 (tracks 4 and 5)

FIG. 6.—The effect of hydroxyurea (HU) and 2′-deoxy-5-azacytidine (daz C) on HSP induction. Track 2: cultures of HeLa cells were treated with 2mM HU for 1 h before normal hyperthermia at 45°C for 5 min, development at 37° for 2 h and labelling with $^{35}$S-methionine for 1 h at 37°C. Track 4 was treated with 1mM dazC for 6 h, before being held at 37°C before normal hyperthermia at 45°C for 5 min followed by development for 2 h and labelling with $^{35}$S-methionine for 1 h at 37°C. The labelled proteins were separated by dodecyl sulphate PAGE and visualised by fluorography. Tracks 3 and 5 received chemicals only, track 1, no treatment.

*The "development" phase of heat-shock protein induction*

At present it is difficult to be precise about events in the 'development' phase. When HeLa cells are heated at 45°C the cellular capacity for incorporation of $^{35}$S-methionine into protein declines (Fig. 7). Analysis of the proteins made under these conditions, however, provides no evidence for induction of HSP synthesis,

FIG. 7.—Effect of "development" at 37°C on amino acid incorporation into protein after hyperthermia. HeLa cells were held at 45°C and labelled with $^{35}$S-methionine for 10 min periods at various times (●). Other cultures were removed after 10 min at 45°C and returned to 37°C (□) or then labelled with $^{35}$S-methionine at 37°C with 4 μg/ml actinomycin D (△) for 10 min periods at times indicated. After labelling, the medium was removed and the level of $^{35}$S incorporation into protein determined as described in Fig. 1.

it appears likely that the methylation status of the genes involved may be such that any further reduction in 5-methyl cytosines is immaterial. This is not surprising, considering the short time-scale involved in HSP induction, compared with metallothionein induction. HSP genes may well be sufficiently undermethylated normally to permit rapid induction.

even over the first 10 min at that temperature. However, if after 10 min the cells are returned to 37°C, there is a marked rapid recovery of amino acid incorporation. In fact, after 45 min at 37°C, the level is higher than in untreated cells (Fig. 7). This recovery is not observed if actinomycin D is added immediately after the 45°C treatment. Whilst such data might suggest a link between the recovery of amino acid incorporation and the induction of HSPs, the recovery process clearly occurs much more rapidly than maximum production of HSPs, which requires at least 2 h (Slater *et al.*, 1981). It may simply be that transcription is required for the recovery process. Goldstein & Penman (1973) suggested that the recovery of protein synthesis, albeit from the lower temperature of 42°C, may be mediated through a short-lived RNA (rather than protein) that promotes the initiation of translation. Recent data of Bonanou-Tzedaki *et al.* (1981) indicate the production of an inhibitor in post-ribosomal supernatants of reticulocyte lysates by brief heat treatment at 44°C. This inhibitor appears identical to the haem-activated inhibitor which phosphorylates the small subunit of the initiation factor elF-2, thereby reducing its catalytic activity. Whether it is this event that could be reversed by an RNA (or an HSP) remains to be answered.

Transcription in HeLa cells is also known to be affected by hyperthermia, though the effects are varied (Zieve *et al.*, 1977). At 43°C tRNA and 5S RNA synthesis remain unaffected, hnRNA and mRNA are still produced, though at reduced rates, but ribosomal RNA is totally inhibited. Whilst recovery during "development" is observed it is clearly

FIG. 8.—The subcellular distribution of HSP in HeLa cells. HeLa cells were labelled for 1 h with L-$^{35}$S-methionine at 37°C, homogenised, and fractionated into various crude subcellular fractions. The pattern of labelled protein associated with each subcellular fraction was determined by fluorography of a dodecyl sulphate/polyacrylamide slab gel. Normal control cells (C) and cells heated at 45° for 10 min followed by 2 h recovery at 37°C before labelling (HS) were fractionated. Cell fractions were: nuclear (nuc) cytoplasmic (cyt) mitochondrial (mit) and post-mitochondrial supernatant (pms).

complex. We have made a preliminary analysis of the situation using cloned cDNA as a probe for specific HeLa 72-74Kd HSP mRNA sequences (Cato *et al.*, 1981). During the "development" phase such sequences can be detected in nuclei by "Northern" blotting of HMW nuclear RNA species, but only after 1–2 h of "development" at 37°C (Cato *et al.*, 1981).

*Intracellular location of HeLa heat-shock proteins*

In a search for pointers to possible function of HeLa HSPs, subcellular fractions were prepared from control and heat-shocked cells labelled with $^{35}$S-methionine. From densitometric analyses of the fluorograms displayed in Fig. 8, it was estimated that whilst only ~30% of the major 72-74Kd group of HSPs are found in the nuclear fraction, 70% remained in the cytoplasm (even after a pulse chase). The 37Kd group occurred in the nucleus as well as the cytoplasm, whereas the 100Kd group appeared exclusively cytoplasmic.

The nuclear HSPs were, furthermore, not removed by 1% Tween-40 or 0·5% sodium deoxycholate, and on further fractionation of the nuclei by the methods of Pederson (1974) were found in association with both chromatin and heterogeneous ribonucleoprotein components. Since the role of the small proportion of HSPs that do associate with the nucleus could be involved in some interaction with

Fig. 9.—The DNA-binding activity of HeLa cell proteins, separated by dodecyl sulphate PAGE and transferred to nitrocellulose filters. $^{35}$S-methionine-labelled protein from control cells (1) and heat-shock cells (2) were "blotted" on to one filter which was impregnated with PPO for fluorography. The remaining tracks of the slab gel (3–10) containing labelled proteins were blotted onto a separate filter, and the filter-bound proteins were tested for their ability to bind $^{32}$P-labelled HeLa DNA. The pattern of DNA binding was visualised by autoradiography of the dried filter. The proteins were prepared from control cells (3) or cells heated at 45°C for 10 min and then allowed to recover at 37°C for:

| Track | 4 | 5 | 6 | 7 | 8 | 9 | 10 |
|---|---|---|---|---|---|---|---|
| Min | 0 | 5 | 10 | 20 | 30 | 60 | 120 |

the genome, the DNA-binding capacity of proteins from heat-shocked cells was assessed by protein "blotting". Proteins, electrophoretically separated on dodecyl sulphate polyacrylamide gels, were transferred to nitrocellulose and tested for ability to bind to HeLa $^{32}$P-DNA. Although several DNA-binding proteins were revealed (Fig. 9) none of them correspond to the HeLa HSPs in the nucleus (*i.e.* 72–74Kd or 37Kd). This of course does not rule out a role for these proteins in some form of transcriptional regulation. A sizeable proportion of *Drosophila* HSPs can also be found associated with chromatin (Velazquez *et al.*, 1980), though recent data (Sinibaldi & Morris, 1981) suggest that they are structural elements.

*Cytoplasmic heat-shock proteins*

Since some 70% of the 72–74Kd proteins, as well as all of the 100 Kd group, appear to be cytoplasmic, further fractionation was carried out. From Fig. 9 it can be seen that they are mainly located in the post-mitochondrial supernatant. A mitochondrial role for HSPs in *Drosophila* was suggested on the basis of the effects of various inhibitors of electron transport and oxidative phosphorylation (Ashburner & Bonner, 1979) but, we find that the following agents failed to induce HSP synthesis in HeLa cells: sodium azide (3 mM), KCN (1 mM) atractyloside (0·1 mM) dinitrophenol (1 mM) sodium arsenate (0·05 mM).

It has also been suggested (Kelley & Schlesinger, 1978) that HSPs are proteins of the cell membrane and possibly involved in hexose transport. However, analysis of plasma-membrane fractions from heat-shocked HeLa cells did not reveal any marked enrichment with HSP. Studies on the *in vitro* translation of HSP mRNAs (Kioussis *et al.*, 1981) indicate that the proteins are not initially synthesised as larger precursors. Also, treatment of HeLa cells with tunicamycin has no effect on the electrophoretic mobility of HSPs, indicating no extensive modification with carbohydrate side chains.

A role in hexose metabolism is possible, but treatment with 2-deoxyglucose (50 mM, 24 h) sodium fluoride (10 mM, 20 min) does not induce HSP synthesis in HeLa cells. In addition, we have examined the level of citric-acid-cycle enzymes (pyruvate dehydrogenase and α-ketoglutarate dehydrogenase) in our HeLa cells. The level of these enzymes is extremely low and is unaffected by heat shock. Examination of glycolytic enzyme activity is in progress.

There are clearly some considerable differences between the HSPs of *Drosophila* and humans. Moreover, it may be unrealistic to expect similar roles for HSPs in such a wide range of species as yeast, *Drosophila* and humans, whose HSPs have different molecular weights. In addition there are clear mechanistic distinctions in their expression. In yeast their induction involves the preferential loss of non-HSP mRNAs, whereas in *Drosophila* there appears to be preferential translation of HSP mRNAs (Lindquist, 1981). In HeLa cells we find no evidence for preferential loss of mRNAs, nor for any apparent preferential translation after heat shock, though the possibility cannot be ruled out at present (Kioussis *et al.*, 1981).

A more useful comparison may be between homeotherms. We find that human (HeLa, HT1080) mouse (L-929) and hamster (BHK-21/C13) cells are similar in that 3 major groups of HSPs are inducible: at ~37 Kd, 72–74 Kd and 100 Kd. Recent data of Levinson *et al.* (1980) indicate that avian cells (chick fibroblasts) have 4 groups of HSP. Two of the avian groups have approximate molecular weights of 70 Kd and 100 Kd and thus probably correspond to the two HMW groups in HeLa cells. Levinson *et al.* (1980) indicates that this correspondence actually extends to similarities revealed by partial proteolytic mapping. However, it must be pointed out that there is some disagreement in the literature concerning the molecular weight of the heaviest group of HSP common to avian and mammalian cells (estimates range from 89 Kd to 100 Kd; see Kelley & Schlesinger, 1978; Levinson *et al.*, 1980; Johnston *et al.*, 1980; Slater *et al.*, 1981; Oppermann *et al.*, 1981; Brugge *et al.*, 1981). Nevertheless, recent interest has focussed on this group, as data now available indicate a possible overlapping role for them in viral oncogenesis and in the heat-shock response. A single viral protein (pp60$^{src}$) mediates the neoplastic transformation of avian cells infected with Rous sarcoma virus. Immunoprecipitation of pp60$^{src}$ has revealed two cellular proteins to be associated with pp60$^{src}$ in a specific manner. One of these belongs to the above-mentioned group of avian HSP (Oppermann *et al.*, 1981; Brugge *et al.*, 1981). Whether any of the corresponding human HSPs play a similar role in human tissues is of course an open question. However, such information would clearly influence hyperthermic protocols in cancer therapy.

Thanks are due to Dr H. G. Nimmo of this Department for assistance with citric-acid-cycle enzyme assays, and to Mrs Vera Holmwood for expert technical assistance.

### REFERENCES

ASHBURNER, M. & BONNER, J. J. (1979) The induction of gene activity in *Drosophila* by heat shock. *Cell*, **17**, 241.

BONANOU-TZEDAKI, S. A., SOHL, M. K. & ARNSTEIN, H. R. V. (1981) Regulation of protein synthesis in reticulocyte lysates: Characterisation of the inhibitor generated in the post-ribosomal supernatant by heating at 44°C. *Eur. J. Biochem.*, **114**, 69.

BOWEN, B., STEINBERG, J., LAEMMLI, U. K. & WEINTRAUB, H. (1981) The detection of DNA-binding proteins by protein blotting. *Nucleic Acids Res.*, **8**, 1.

BRUGGE, J. S., ERIKSON, E. & ERIKSON, R. L. (1981) The specific interaction of the Rous sarcoma virus transforming protein pp60$^{src}$ with two cellular proteins. *Cell*, **25**, 363.

CATO, A. C. B., SILLAR, G. M., KIOUSSIS, J. & BURDON, R. H. (1981) Molecular cloning of cDNA sequences coding for the major ($\beta$, $\gamma$, $\delta$ and $\epsilon$) heat shock polypeptides of HeLa cells. *Gene*, **16**, 27.

COMPERE, S. J. & PALMITER, R. D. (1981) DNA methylation controls the inducibility of the mouse metallothionein-1 gene in lymphoid cells. *Cell*, **25**, 233.

CONNER, W. G., GARNER, E. W., MILLER, R. C. & BOONE, M. L. M. (1977) Prospects for hyperthermia in human cancer therapy. II. Implications of biological and physical data for application of hyperthermia to man. *Radiology*, **123**, 497.

DEWEY, W. C., HOPWOOD, L. E., SAPORETO, S. A. & GERWECK, L. E. (1977) Responses to combinations of hyperthermia and radiation. *Radiology*, **123**, 463.

FRASER, N. W., BURDON, R. H. & ELTON, R. A. (1975) Comparison of nucleotide sequences in HeLa cell mRNA and hnRNA. *Nucleic Acids Res.*, **2**, 2131.

GERNER, E. W., BOONE, R., CONNOR, W. G., HICKS, J. A. & BOONE, M. L. M. (1976) A transient thermotolerant survival response produced by single thermal doses in HeLa cells. *Cancer Res.*, **36**, 1035.

GOLDSTEIN, E. S. & PENMAN, S. (1973) Regulation of protein synthesis in mammalian cells. V. Further studies on the effect of actinomycin D on translation control in HeLa cells. *J. Mol. Biol.*, **80**, 243.

HAR-KEDAR, I. & BLEEHEN, N. M. (1976) Experimental and clinical aspects of hyperthermia applied to the treatment of cancer with special reference to the role of ultrasonic and microwave heating. *Adv. Radiat. Biol.*, **6**, 229.

HENLE, K. & DETHLEFSEN, L. A. (1978) Heat fractionation and thermotolerance: A review. *Cancer Res.*, **38**, 1843.

HENLE, K. J., KARAMUZ, J. E. & LEEPER, D. B. (1978) Induction of thermotolerance in Chinese hamster ovary cells by high (45°) and low (40°) hyperthermia. *Cancer Res.*, **38**, 570.

JOHNSEN, S., STOKKE, T. & PRYDZ, H. (1974) HeLa cell plasma membranes. I. 5′-Nucleotidase and oubain sensitive ATPase as markers for plasma membranes. *J. Cell. Biol.*, **63**, 357.

JOHNSTON, D., OPPERMAN, H., JACKSON, J. & LEVINSON, W. (1980) Induction of four proteins in chick embryo cells by sodium arsenite. *J. Biol. Chem.*, **255**, 6975.

JONES, P. A. & TAYLOR, S. M. (1980) Cellular differentiation, cytidine analogues and DNA methylation. *Cell*, **20**, 85.

KELLEY, P. M. & SCHLESINGER, M. J. (1978) The effect of amino acid analogues and heat shock on gene expression in chicken embryo fibroblasts, *Cell*, **15**, 1277.

KIOUSSIS, J., CATO, A. C. B., SLATER, A. & BURDON, R. H. (1981) Polypeptides encoded by polyadenylated and non-polyadenylated messenger RNAs from normal and heat shocked HeLa cells. *Nucleic Acids Res.*, **9**, 5203.

LEPOCK, J. R. & KRUUV, J. (1980) Thermotolerance as a possible cause of the critical temperature at 43° in mammalian cells. *Cancer Res.*, **40**, 4485.

LEVINSON, W., OPPERMANN, H. & JACKSON, J. (1980) Transition series metals and sulphydryl reagents induce the synthesis of four proteins in eukaryotic cells. *Biochim. Biophys. Acta*, **606**, 170.

LINDQUIST, S. (1981) Regulation of protein synthesis during heat shock. *Nature*, **293**, 311.

MILLER, R. C., CONNOR, W. G., HEUSENHUELD, R. S. & BOONE, M. L. M. (1977) Prospects for hyperthermia in human cancer therapy. I. Hyperthermic effects in man and spontaneous animal tumours. *Radiology*, **123**, 489.

OPPERMANN, H., LEVINSON, W. & BISHOP, J. M. (1981) A cellular protein that associates with the transforming protein of Rous sarcoma virus is also a heat-shock protein. *Proc. Natl Acad. Sci.*, **78,** 1067.

OVERGAARD, J. & BICHEL, P. (1977) The influence of hypoxia and acidity on the hyperthermic response of malignant cells *in vitro. Radiat. Biol.*, **123,** 511.

PEDERSON, T. (1974) Proteins associated with heterogeneous nuclear RNA in eukaryotic cells. *J. Mol. Biol.* **83,** 163.

PENMAN, S. (1969) Preparation of nuclei and nucleoli from mammalian cells. In *Fundamental Techniques in Virology* (Eds. Habel & Salzman) New York: Academic Press. p. 35.

RIGBY, P. W. J., DIECKMAN, M., RHODES, C. & BERG, P. (1977) Labelling DNA to high specific activity *in vitro* by nick translation with DNA polymerase 1. *J. Mol. Biol.*, **113,** 237.

ROSENBERG, B., KEMENY, G., SWITZER, R. C. & HAMILTON, T. C. (1971) Quantitative evidence for protein denaturation as the cause of thermal death. *Nature*, **232,** 471.

SINIBALDI, R. M. & MORRIS, P. W. (1981) Putative function of *Drosophila melanogaster* heat shock proteins in the nucleoskeleton. *J. Biol. Chem.*, **256,** 10735.

SLATER, A., CATO, A. C. B., SILLAR, G. M., KIOUSSIS, J. & BURDON, R. H. (1981) The pattern of protein synthesis induced by heat shock of HeLa cells. *Eur. J. Biochem.*, **117,** 341.

SUIT, H. D. (1977) Hyperthermia effects on animal tissues. *Radiology*, **123,** 483.

VELAZQUEZ, J. M., DIDOMENICO, B. J. & LINDQUIST, S. (1980) Intracellular localisation of heat shock protein in *Drosophila. Cell*, **20,** 679.

ZIEVE, G., BENECKE, B. & PENMAN, S. (1977) Synthesis of two classes of small RNA species *in vivo* and *in vitro. Biochemistry*, **16,** 4520.